# Supplementary material for: Automated Deep Learning Pipeline for Callosal Angle Quantification
Source: medRxiv. 2025 Aug 21:2025.08.18.25333901. Preprint. [Version 1] doi: 10.1101/2025.08.18.25333901 (PMC12393603; doi:10.1101/2025.08.18.25333901)
Supplement: Supplement 1 [file media-1.docx]

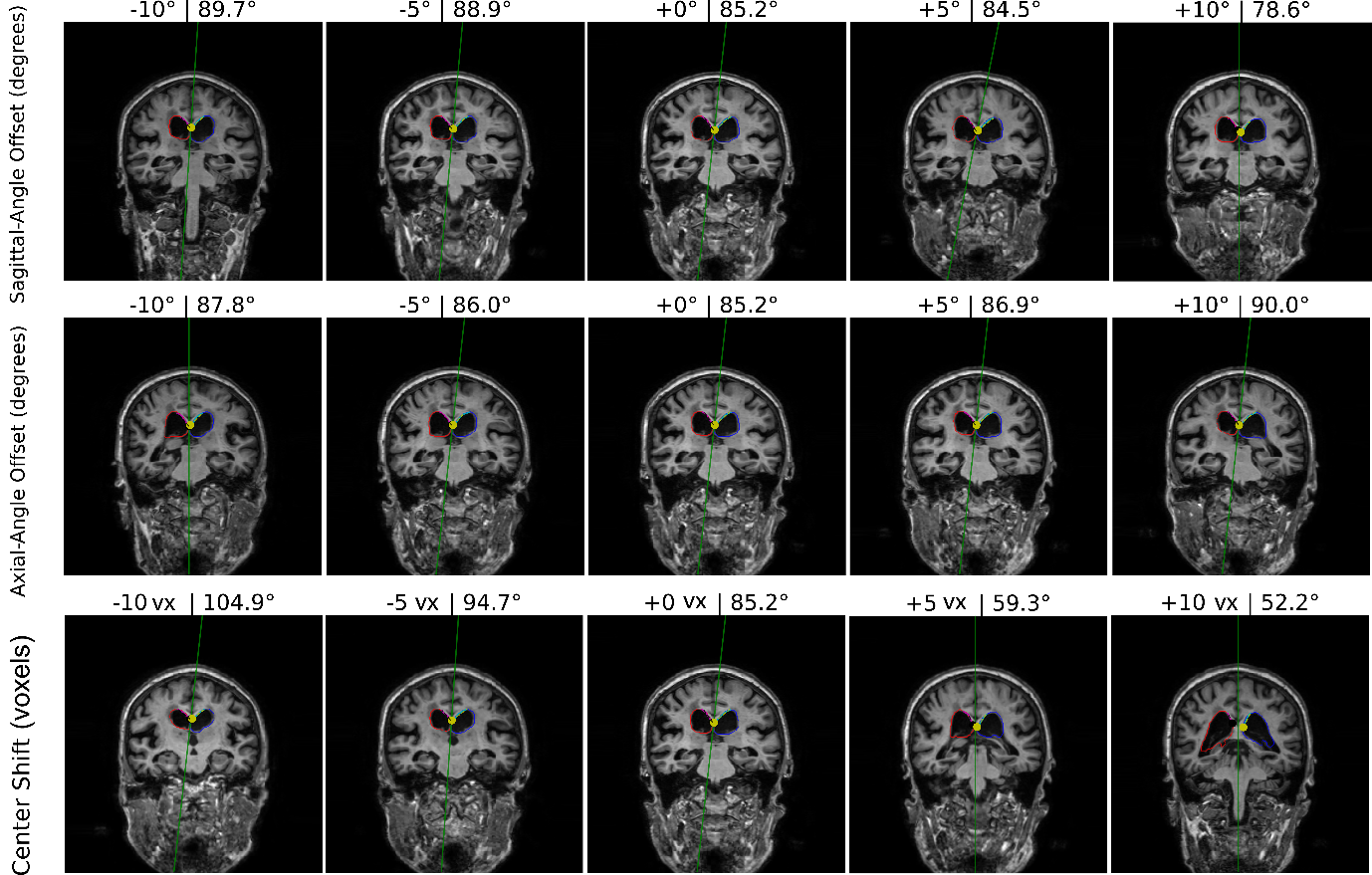


Supplementary Figure 1. Coronal‐plane for one subject under three perturbation types: sagittal‐plane rotations, axial‐plane rotations, and PC landmark shifts. Each row shows five steps (−10, −5, 0, +5, +10) of bias in angles (degree) and location (mm). As well as calculated Callosal Angle (degree).
