# Supplementary material for: Automated Deep Learning Pipeline for Callosal Angle Quantification
Source: medRxiv. 2025 Aug 21:2025.08.18.25333901. Preprint. [Version 1] doi: 10.1101/2025.08.18.25333901 (PMC12393603; doi:10.1101/2025.08.18.25333901)
Supplement: Supplement 2 [file media-2.docx]

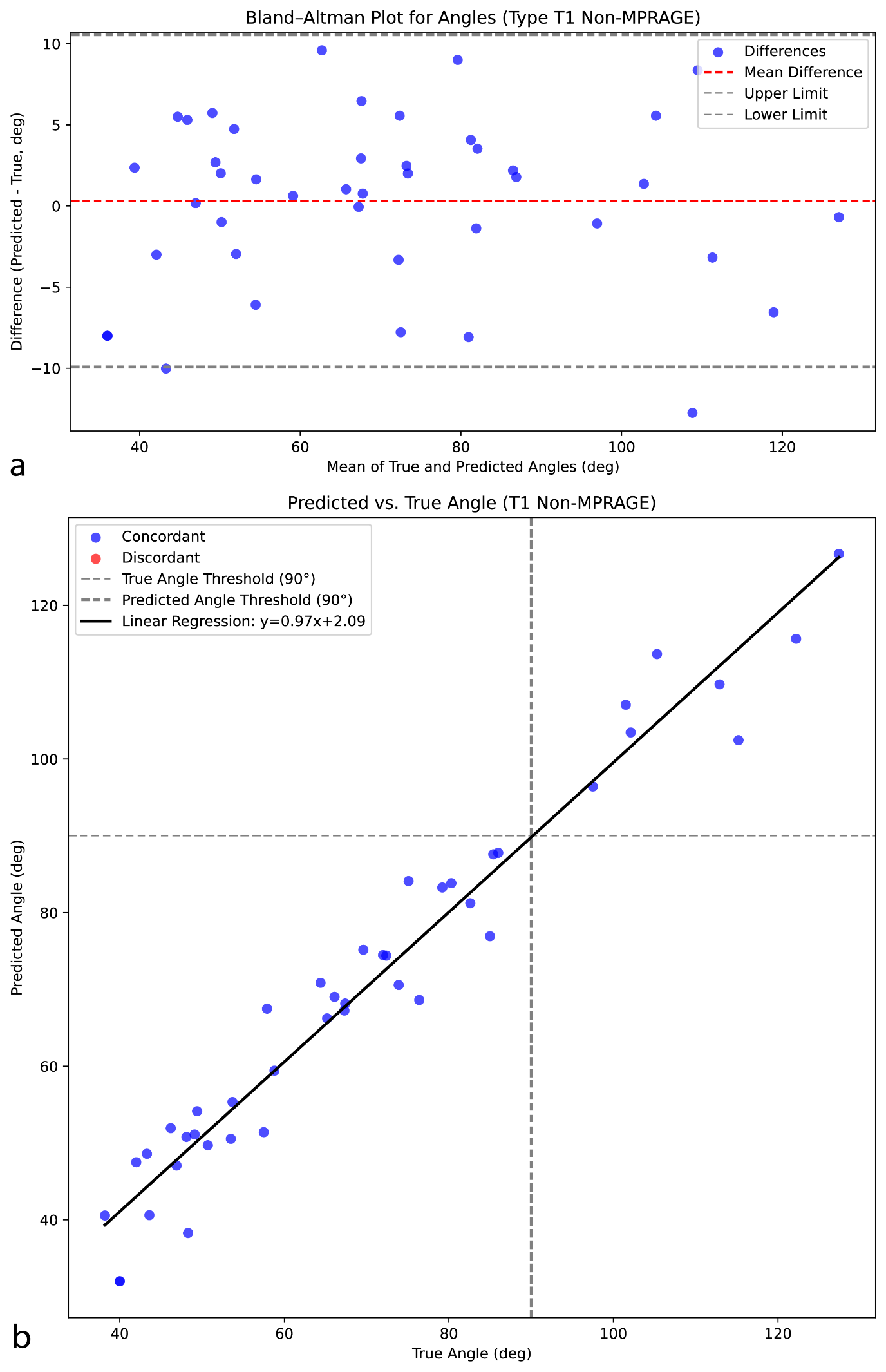


Supplementary Figure2. Overview of the evaluation of callosal angle (CA) measurements on non-MPRAGE scans. Panel (a) displays a Bland-Altman plot comparing the predicted CA values with the manual measurements obtained from corresponding MPRAGE scans. Panel (b) illustrates the correlation between the predicted and manually annotated CA values via a scatter plot.
